# Characterization of embryonic stem cell-differentiated fibroblasts as mesenchymal stem cells with robust expansion capacity and attenuated innate immunity

**DOI:** 10.1186/s13287-018-1033-8

**Published:** 2018-10-25

**Authors:** William D’Angelo, Bohan Chen, Chandan Gurung, Yan-Lin Guo

**Affiliations:** Department of Cell and Molecular Biology, University of Southern Mississippi, 118 College Drive 5018, Hattiesburg, MS 39406 USA

**Keywords:** Embryonic stem cells, Mesenchymal stem cells, Differentiation, Cell cycle, Senescence, Immunosuppression, Inflammatory cytokines, Tissue regeneration

## Abstract

**Background:**

Mesenchymal stem cells (MSCs) isolated from adult tissues (Ad-MSCs) have shown great promise for use in regenerative medicine. However, their poor in vitro expansion capacity and tissue scarcity have been major limitations. In this study, we demonstrate that mouse embryonic stem cells (mESCs) can differentiate into cells with MSC properties.

**Methods:**

Using previously established methods that characterize Ad-MSCs, we analyzed mESC-differentiated fibroblasts (mESC-FBs), including plastic adherence, clonogenic growth, MSC marker expression, tri-lineage differentiation potential, and the capacity to express immunomodulators.

**Results:**

Although previously characterized as mESC-differentiated fibroblasts (mESC-FBs), these cells exhibit major properties of Ad-MSCs. However, mESC-FBs also display unique features inherited from ESCs, including robust expansion capacity, senescence resistance, and attenuated innate immunity. In particular, mESC-FBs are insensitive to bacterial endotoxin (lipopolysaccharide, LPS) and do not express LPS-induced inflammatory molecules, in contrast to bone marrow (BM)-MSCs. We further demonstrate that mESC-FBs are resistant to the cytotoxicity associated with inflammatory cytokines, bacterial endotoxins (LPS and heat-killed bacteria), and macrophage-mediated inflammation.

**Conclusions:**

While it remains to be determined how the unique properties of mESC-FBs will affect their immunoregulatory activity under an in vivo condition, our findings demonstrate that ESCs could be used as an alternative source to generate a new class of ESC-MSCs with unique features potentially useful in regenerative medicine.

## Background

Mesenchymal stem cells (MSCs**)** isolated from adult tissues (Ad-MSCs) have rapidly advanced to clinical application due to their defined differentiation potential (typically to osteocytes, chondrocytes, and adipocytes) and their ability to secrete immunomodulators and trophic growth factors that repress inflammation and promote tissue regeneration in the host [1–3]. However, limited availability, low expansion capacity, and significant donor-dependent variations have been the major limitations for their therapeutic application [1, 4]. On the other hand, embryonic stem cells (ESCs) have greater potential to differentiate into a wide variety of cell types and have unlimited expansion capacity [5], but generating clinically usable cells from ESCs is a task facing many biological and technical challenges [6], including uncertainty about the physiology, maturity, and functionality of in vitro ESC-differentiated cells (ESC-DCs) owing to their dramatically different routes of differentiation from naturally differentiated somatic cells.

The underdeveloped innate immunity in ESCs suggests that “innate immunity,” as classically defined in somatic cells, is not (at least not completely) innate to ESCs, but is acquired by somatic cells during differentiation, as we have demonstrated in mouse ESC (mESC) differentiation to fibroblasts (mESC-FBs) [7–9]. FBs are highly responsive to various immune stimuli and are a major tissue cell type responsible for maintaining tissue immunity [10, 11]. Using mESC-FBs as a model system, we investigated innate immunity development during in vitro differentiation and demonstrated that mESC-FBs acquired the ability to express IFNβ and inflammatory cytokines in response to viral stimuli and TNFα, respectively; however, they have substantially lower levels of responsiveness than naturally differentiated FBs [7–9]. Together with similar results found in other types of ESC-DCs [12–16], our findings demonstrated that innate immunity is developmentally regulated, but the commonly used in vitro differentiation methods can only partially promote this development.

Originally isolated and characterized as FB-like multipotent cells from bone marrow [2], bone marrow-MSCs (BM-MSCs) can differentiate into adipocytes, osteocytes, or chondrocytes (known as tri-lineage differentiation). This property is regarded as the basis of using BM-MSCs in tissue repair and regeneration, but it is now increasingly clear that their therapeutic effects are largely attributable to their ability to secrete immunoregulators and trophic factors (collectively referred to as “bioactive factors”), which modulate host immunity and promote tissue healing via paracrine signaling [3]. MSCs have now been isolated from a variety of adult and fetal tissues. Interestingly, studies have shown that MSCs share many similarities with FBs, for instance in morphology and cell-surface markers. In fact, BM-MSCs were first isolated based on their plastic-adherence property originally noted in FBs. Furthermore, immunoregulatory properties similar to MSCs have been reported in adult human FBs [17, 18]. While the exact relationship between FBs and MSCs remains to be defined, existing evidence suggests that they may represent subtypes of the same (or similar) cells with overlapping functions [17, 19, 20]. While using mESC-FBs as a model to study innate immunity development, we have noticed that they display the major characteristics of Ad-MSCs. In this study, we demonstrate that mESC-FBs (as we previously designated them) can also be classified as mESC-MSCs. Although ESC-MSCs have been differentiated from both mESCs and human ESC (hESCs) as well as iPSCs and compared with Ad-MSCs in terms of basic MSC properties and therapeutic potential [21], they are not nearly as well-characterized as Ad-MSCs. In particular, their attenuated innate immunity inherited from ESCs, together with their robust expansion capacity and resistance to senescence, can classify ESC-MSCs as a new type of MSCs with valuable attributes for therapeutic application.

## Methods

### Cells and cell culture

mESC-FBs were differentiated from D3 and DBA252 mESCs through a retinoic acid (RA)-induced differentiation protocol and purified based on their rapid adherence to an uncoated cell culture dish as described in our published studies [7, 8]. Since mESC-FBs differentiated from D3 and DBA mESCs were similar in all properties tested, the experiments in this study were mainly performed with mESC-FBs differentiated from D3 cells and some key experiments were confirmed with the cells differentiated from DBA cells. mESC-FBs from passages 5–35 were used for this study. Primary MSCs from mouse bone marrow (BM-MSCs) were obtained from an NIH-funded research center at The Scripps Research Institute – Scripps Florida, Department of Molecular Therapeutics [22]. Cells from passage 2 to 5 were used for the experiments. RAW264.7 cells (RAW, a murine macrophage cell line) were obtained from ATCC (Manassas, VA, USA). All cells were cultured in DMEM with 10% fetal bovine serum (FBS) and 100 units/ml penicillin and 100 μg/ml streptomycin and maintained at 37 °C in a humidified incubator with 5% CO_2_.

### Preparation of conditioned medium and heat killed bacteria and cell treatment

To prepare conditioned medium (CM), RAW cells were treated with lipopolysaccharide (LPS) (1 μg/ml, isolated from *E.coli* O111:B4, Sigma) for 4 h. The medium was removed and cells were thoroughly washed with PBS and then cultured in fresh medium for an additional 24 h. The CM was collected and designated as LPS-CM. CM prepared from RAW cells without treatment was used as a control (CM). Heat-killed *E. coli* (O157:H7, ATCC) (HKE) were prepared by heating bacteria in PBS at 80 °C for 1 h [23]. mESC-FBs and BM-MSCs were treated with CM or LPS-CM (diluted with fresh medium containing 2% FBS at 1:1 ratio), HKE (at a ratio of 200:1 bacteria to mESC-FBs or BM-MSCs), LPS (1 μg/ml), TNFα, IL-1β, or IFNγ (20 ng/ml, Peprotech, Rocky Hill, NJ, USA). For cytotoxicity analysis, cells were treated in DMEM containing 2% FBS. For all other experiments, cells were treated in DMEM containing 10% FBS under the conditions as specified in the individual experiments.

### Analysis of cell proliferation, viability, clonal growth, and senescence

Cell proliferation and viability were determined by toluidine blue (TB) staining as previously described [24]. The absorbance at 630 nm of stained cells was measured with a Biotek ELx800 microtiter plate reader. The absorbance values were used as an indirect measurement of cell number, or the numbers of cells were counted from photographed images of TB stained cells as described in individual experiments. For clonal growth analysis, mESC-FBs were plated in a six-well cell culture dish at low density (~ 400 cells/well) and allowed to grow for 2 weeks. The colonies derived from single cells were fixed and stained with TB for morphological analysis or further propagated to determine cell growth rate. Senescence was determined by morphological criteria and by cellular/biochemical marker analysis as previously described [24]. Briefly, mESC-FBs and BM-MSCs were plated at ~ 50% confluence and cultured for 7 days. The cells were analyzed with a β-galactosidase (β-Gal) senescence detecting kit (Sigma-Aldrich, St. Louis, MO, USA) and by the expression levels of senescence markers, p21 and p16.

### Tri-lineage differentiation of mESC-FBs and detection of adipocytes, osteocytes, and chondrocytes

The potential of mESC-FBs to differentiate to adipocytes, osteocytes, and chondrocytes was assessed by a spontaneous differentiation protocol. mESC-FBs were seeded at 60–70% confluence and continuously cultured in a dish up to 4 weeks without splitting, during which cells underwent spontaneous differentiation. Many cells in the monolayer developed oil droplets, a characteristic of adipocytes that can be visually detected in live cells. For further analysis, the cells were fixed and stained with 0.5% Oil-Red O, 2% Alizarin Red S, or 0.1% Safranin O (Sigma-Aldrich) to stain adipocytes, osteocytes, or chondrocytes, respectively, according to published protocols [22, 25]. The differentiated cells were further assessed by the expression of adipocyte, osteocyte, and chondrocyte markers with RT-qPCR.

### Real-time quantitative polymerase chain reaction (RT-qPCR)

Total RNA was extracted using TRI-reagent (Sigma-Aldrich). cDNA was prepared using moloney murine leukemia virus reverse transcriptase (Promega, Madison, WI, USA). RT-qPCR was performed using SYBR green ready mix (Bio-Rad) on a MX3000P RT-PCR system (Agilent, Santa Clara, CA, USA). The mRNA levels from RT-qPCR were calculated using the comparative Ct method [26]. β-actin was used as a calibrator for the calculation of relative mRNA levels of the tested genes. As specified in individual experiments, the mRNA levels were either expressed as fold-activation, where the values in the controls were designated as 1, or expressed as relative levels normalized to β-actin (designated as 1). The sequences of the primer sets utilized for RT-qPCR are listed in Table 1.

**Table 1:** The primer sequences used for RT-qPCR

Gene	Forward primer (5′–3′)	Reverse primer (5′–3′)
β-actin	CATGTACGTAGCCATCCAGGC	CTCTTTGATGTCACGCACGAT
Runx2	GCCCAGGCGTATTTCAGA	TGCCTGGCTCTTCTTACTGAG
OCN	CTGACCTCACAGATGCCAAG	GTAGCGCCGGAGTCTGTT
SOX9	AGTACCCGCATCTGCACAAC	ACGAAGGGTCTCTTCTCGCT
Col2α1	GGGTCACAGAGGTTACCCAG	ACCAGGGGAACCACTCTCAC
PPARγ	GGAAGACCACTCGCATTCCTT	GTAATCAGCAACCATTGGGTCA
C/EBPα	CAAGAACAGCAACGAGTACCG	GTCACTGGTCAACTCCAGCAC
CD106	CCAAATCCACGCTTGTGTTGA	GGAATGAGTAGACCTCCACCT
CD105	AGGGGTGAGGTGACGTTTAC	GTGCCATTTTGCTTGGATGC
CD73	CCTGCACACAAACGACGTG	CTGGTCTCCGGCATCCAAAA
CD29	ATGCCAAATCTTGCGGAGAAT	TTTGCTGCGATTGGTGACATT
CD44	TCGATTTGAATGTAACCTGCCG	CAGTCCGGGAGATACTGTAGC
CD31	TGCACCCATCACTTACCACC	CTTCATCCACCGGGGCTATC
CD34	CTGGGTAGCTCTCTGCCTGAT	TGGTAGGAACTGATGGGGATATT
TNFα	CAGGCGGTGCCTATGTCTC	CGATCACCCCGAAGTTCAGTAG
MHCI	CCTTGGAGCTGCAATAGTC	CTGGGAGAGACAGATCAGAG
MHCII	CAACCGTGACTATTCCTTCC	CTCAGGTTCCCAGTGTTTC
iNOS	CAGCACAGGAAATGTTTCAGC	TAGCCAGCGTACCGGATGA
CyclinD1	CAGAAGTGCGAAGAGGAGGTC	TCATCTTAGAGGCCACGAACAT
CyclinE	AATTGGGGCAATAGAGAAGAGGT	TGGAGCTTATAGACTTCGCACA
CyclinA	ACATTCACACGTACCTTAGGGA	CATAGCAGCCGTGCCTACA
CyclinB	CTCAGGGTCACTAGGAACACG	AGCTCTTCGCTGACTTTATTACC
P16	CGCAGGTTCTTGGTCACTGT	TGTTCACGAAAGCCAGAGCG
P21	CGAGAACGGTGGAACTTTGAC	CAGGGCTCAGGTAGACCTTG
VEGF	GGAGATCCTTCGAGGAGCACT	GGCGATTTAGCAGCAGATATAA
SCF	CCCTGAAGACTCGGGCCTA	CAATTACAAGCGAAATGAGAGCC
TGFβ	ATCCTGTCCAAACTAAGGCTCG	ACCTCTTTAGCATAGTAGTCCGC
PDGF	CATCCGCTCCTTTGATGATCTT	GTGCTCGGGTCATGTTCAAGT
HGF	ATGTGGGGGACCAAACTTCTG	GGATGGCGACATGAAGCAG
SDF	TGCATCAGTGACGGTAAACCA	CACAGTTTGGAGTGTTGAGGAT
CTGF	GGGCCTCTTCTGCGATTTC	ATCCAGGCAAGTGCATTGGTA
bFGF	GCGACCCACACGTCAAACTA	CCGTCCATCTTCCTTCATAGC
ICAM1	GGCATTGTTCTCTAATGTCTCCG	GCTCCAGGTATATCCGAGCTTC
IL6	TAGTCCTTCCTACCCCAATTTCC	TTGGTCCTTAGCCACTCCTTC
IL1α	TCTATGATGCAAGCTATGGCTCA	CGGCTCTCCTTGAAGGTGA

### Protein analysis by flow cytometry

Protein analysis by flow cytometry was performed according to our published method [27]. Briefly, fixed cells were incubated with the antibodies against the specific proteins to be analyzed, including antibodies for iNOS, COX2 (Santa Cruz Biotechnology, Santa Cruz, CA, USA), CD29, CD44, CD105, and CD31 (BD Biosciences, Billerica, MA, USA) as specified in individual experiments. The cells were then incubated with secondary antibodies conjugated with FITC (fluorescein isothiocyanate) and examined with an Accuri C6 flow cytometer (BD Biosciences, San Jose, CA, USA). The fluorescence intensity, which correlates with the protein level, was determined with the CFlow software [27].

### Immunocytochemistry

Immunostaining was performed according to the method previously described [7]. The cellular location of NFκB was determined with its specific antibodies against RelA subunit (Santa Cruz Biotechnology). The cells were examined under an LSM 510 laser-scanning confocal microscope (Zeiss).

### Statistical analysis

For statistical analysis, data are presented as the mean + SD derived either from three independent experiments or from a representative experiment performed in triplicate that was performed at least twice with similar results. Statistical analysis was performed using a two-tailed and paired Student’s *t* test. Differences are considered statistically significant when *p* < 0.05.

## Results

### Tri-lineage differentiation of mESC-FBs

**Fig. 1:**
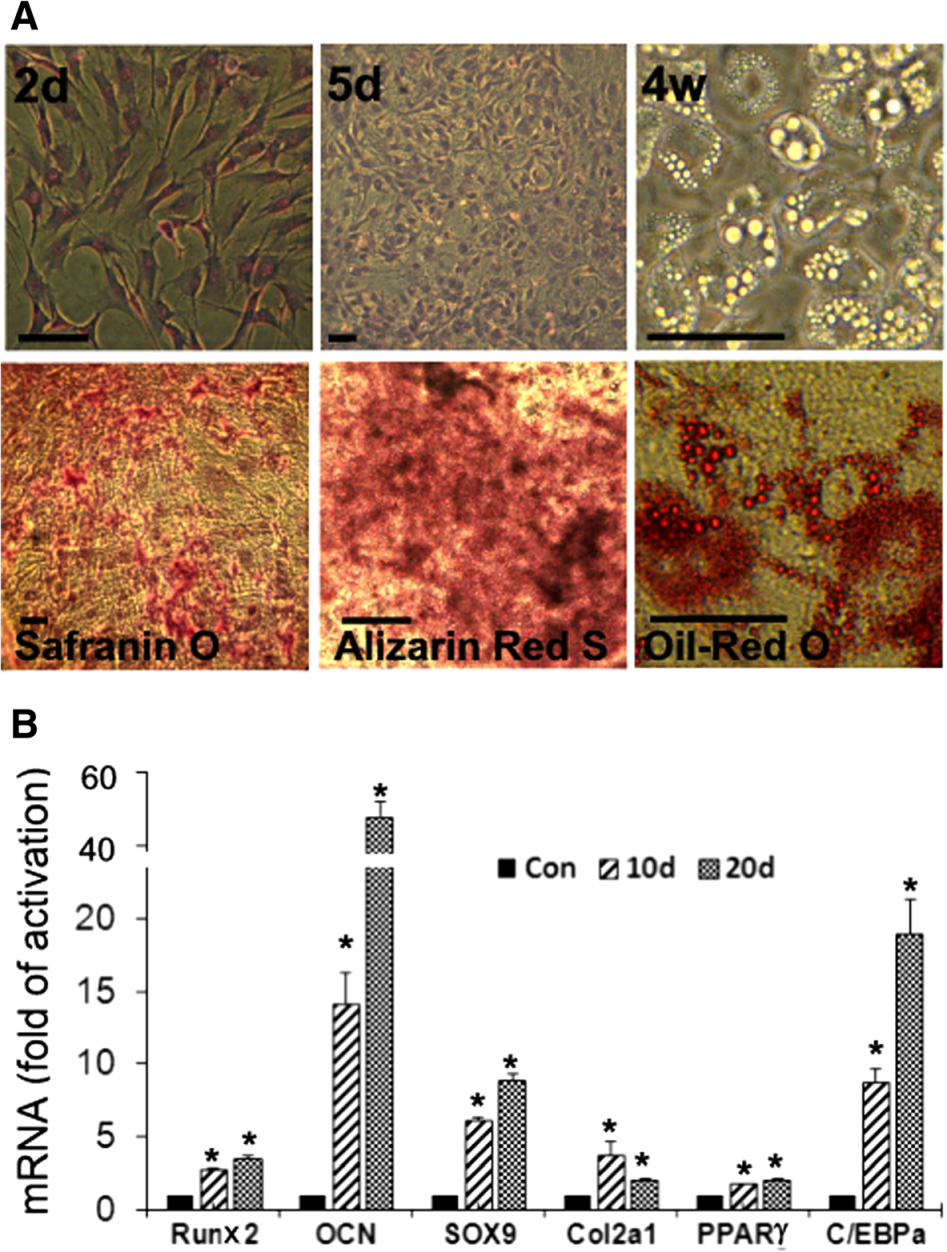
Tri-lineage differentiation of mESC-FBs. Cells were differentiated for up to 4 weeks and were analyzed at the times indicated. **a** Upper panels: 2d, morphology of individual cells at low density after seeding for 2 days; 5d, monolayer formed from cells cultured for 5 days; 4w, oil droplets formed in adipocytes after the cells were cultured for 4 weeks. Bottom panels: cells differentiated for 3 weeks were fixed and stained with the indicated dyes. The cells were photographed under a phase contrast microscope after they were stained with TB (2d and 5d), Oil Red O, Alizarin Red, or Safranin O. The cells shown in 4w panel were live cells without staining. Scale bar = 100 μm. **b** Analysis of tri-lineage marker expression by RT-qPCR in the cells differentiated for 10 days and 20 days. The values in the control group represent the mRNA levels in cells before differentiation. Values are mean ± SD of an experiment performed in triplicate and repeated three times with similar results. *p < 0.05, compared with respective controls (Con)

In our previous studies, we have comparatively analyzed mESC-FBs with mouse embryonic fibroblasts. They show very similar morphology and express common markers of fibroblasts [7–9]. In addition to the complete loss of ESC morphology, the expression level of the pluripotency marker genes Oct4, Nanog, and Sox2 in mESC-FBs is less than 1% of that expressed in mESCs (data not shown). The first indication suggesting that mESC-FBs have the properties of MSCs was their differentiation to adipocytes. As shown in Fig. 1a, mESC-FBs showed typical morphology of FBs and MSCs at sub-confluence (Fig. 1a, 2d). When mESC-FBs were continually cultured, they grew into a compact cell layer and underwent spontaneous differentiation after prolonged culture without passaging (Fig. 1a, 5d). Oil droplets began to appear in some cells around 2 weeks of differentiation and the number and size of these structures increased along with continued culture for up to 4 weeks (Fig. 1a, 4w). These droplets stained heavily with Oil Red O, which is used to identify adipocytes. The differentiated cell layer also contained cells that stained positively with Alizarin Red S and Safranin O (Fig. 1a), two chemical agents commonly used to identify osteogenic and chondrogenic differentiation, respectively. The identities of these cells were confirmed by the expression of their marker genes or transcription factors that promote their differentiation (Fig. 1b, Runx2/OCN for osteocytes, Sox9/COL2A1 for chondrocytes, and PPARγ/C/EBPα for adipocytes). These results demonstrate that mESC-FBs have the potential for tri-lineage differentiation shown by Ad-MSCs.

### mESC-FBs express common MSC markers and have clonal growth capacity

**Fig. 2:**
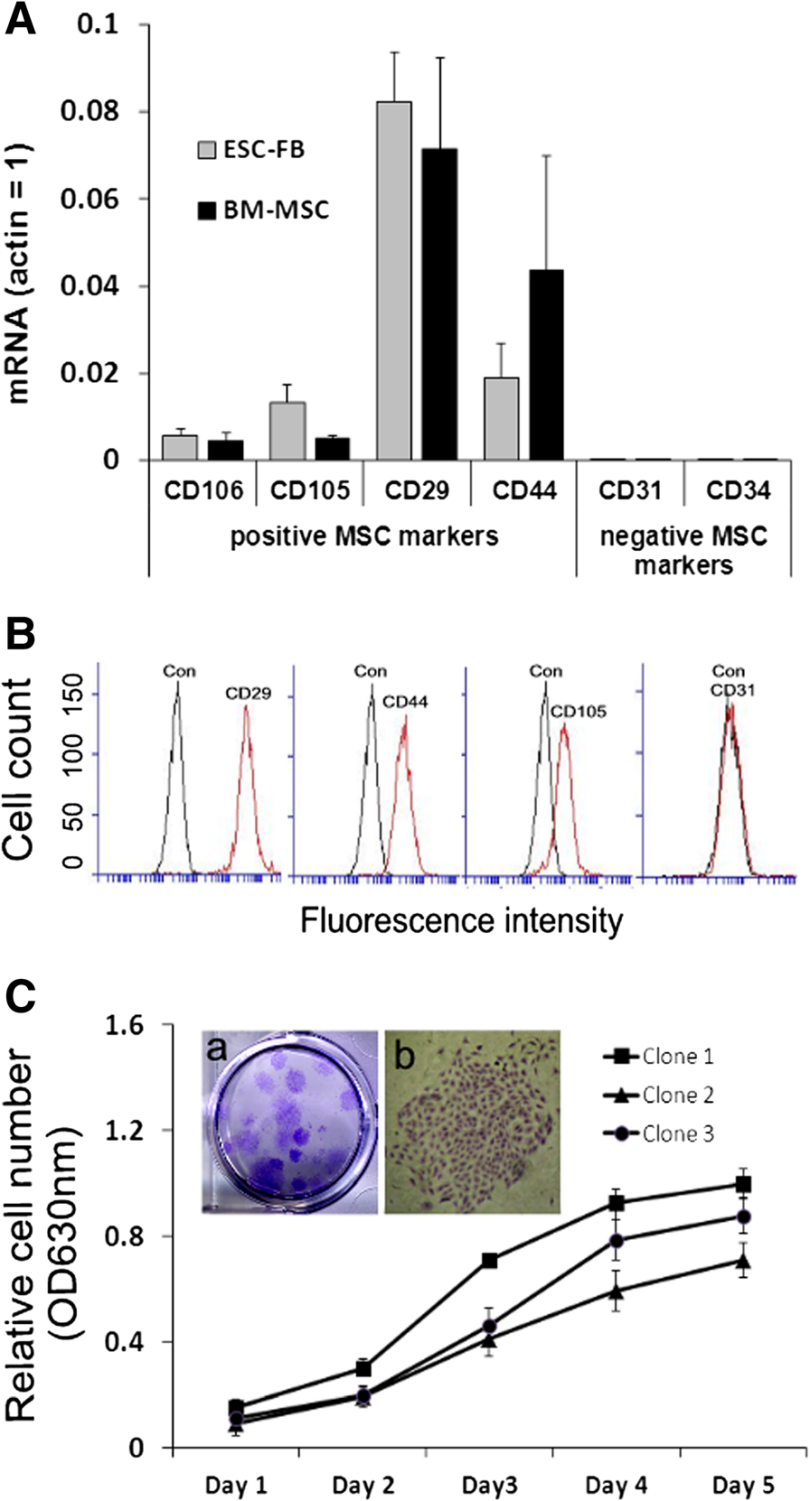
MSC marker expression and clonal growth of mESC-FBs. **a** Comparative analysis of MSC marker expression by RT-qPCR in mESC-FBs and BM-MSCs. Results are mean ± SD of three independent experiments. **b** Flow cytometry analysis of selected MSC markers in mESC-FBs. Con represent cells stained with isotype control antibodies. Results are representatives of three independent experiments. **c** Clonal growth of mESC-FBs. The cells in the plate were photographed with a digital camera (a) or cells in a single colony photographed under a phase contrast microscope (100×) after they were stained with TB. The graph illustrates growth curves of three representative clones. Values are mean ± SD of an experiment performed in triplicate and repeated two times with similar results

While there are no definitive markers that can reliably define MSCs, they are generally characterized by the lack of hematopoietic and endothelial markers and positive expression of a panel of surface markers [20, 28]. We tested markers that are commonly used to identify Ad-MSCs, including CD29, CD44, CD105, and CD106. All of them were detected in mESC-FBs with a very similar expression pattern to BM-MSCs at the mRNA level while CD31 and CD34 (endothelial and hematopoietic markers, respectively) were not detected (Fig. 2a). The results were confirmed by the expression of selected markers at the protein level by flow cytometry (Fig. 2b). When seeded at very low density, single mESC-FBs grew into individual colonies of FB-like clonogenic progeny (known as colony-forming unit FBs). This assay was originally used to determine the number of BM-MSCs within a given cell population of bone marrow cells and is considered to be a major parameter of self-renewal capacity of MSCs [29]. Single mESC-FBs grew into individual colonies of various sizes (Fig. 2c, inset, a). The cells within a colony have fairly uniform morphology, as shown in a representative colony (Fig. 2c, inset, b), although cells from different colonies showed certain degrees of differences in morphology (data not shown) and growth rate (Fig. 2c, graph).

### mESC-FBs can express immune modulators in response to inflammatory cytokines and have low levels of expression of the major histocompatibility complexes (MHCs)

**Fig. 3:**
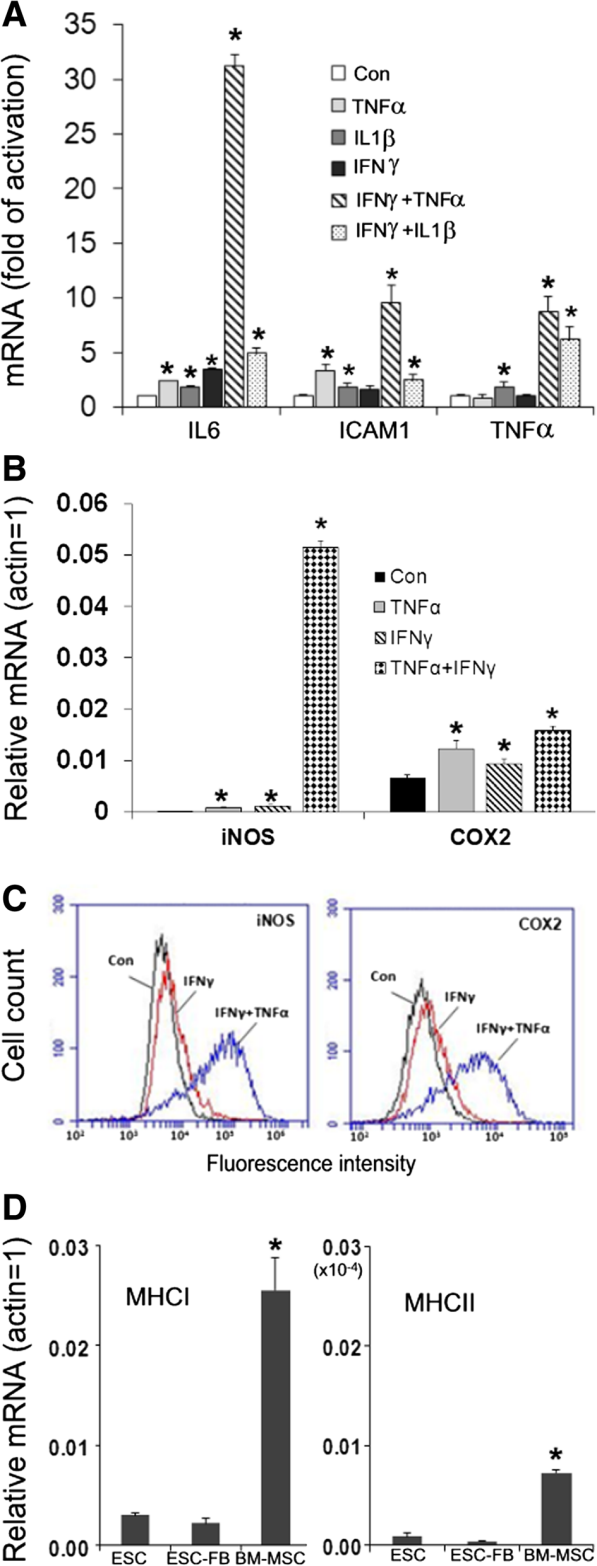
Immunologic properties of mESC-FBs. **a** Expression of immunomodulators induced by inflammatory cytokines. Cells were treated for 24 h with indicated cytokines, alone or in combination. The expression levels of IL6, TNFα and ICAM1 were determined by RT-qPCR analysis. **b**, **c** Analysis of IFNγ- and TNFα-induced iNOS and COX2 expression by RT-qPCR (**b**) and flow cytometry (**c**). **d** Comparative analysis of MHC expression in BM-MSCs, mESCs and mESC-FBs by RT-qPCR. Values in RT-PCR analysis are mean ± SD of data from three independent experiments. *p < 0.05, **a**, **b**, compared with respective controls (Con); **d** compared with ESC. The results in flow cytometry are representative experiments performed three times with similar results

In response to immune and inflammatory stimuli, MSCs produce cytokines and immune modulators, which modulate tissue immunity and activities of immune cells [1, 3]. We evaluated the response of mESC-FBs to IFNγ, TNFα, and IL1β, three cytokines known to activate BM-MSCs [30]. Each cytokine alone slightly stimulated (two- to fourfold) the expression of IL6, ICAM1, and TNFα (common effectors of inflammatory responses), but the combination of IFNγ with TNFα, and to a lesser extent, with IL1β had significant synergistic effects in stimulating the expression of these molecules (Fig. 3a). Similar results were observed for inducible nitric oxide synthase (iNOS) and cyclooxygenase-2 (COX2) as determined at both mRNA and protein levels in mESC-FBs (Fig. 3b, c), two key molecules in mediating the immunomodulatory effects of mouse BM-MSCs [21, 30].

The immune tolerance of BM-MSCs in cell transplant is, in part, due to the low level expression of MHCs that allow them to avoid the host’s immune rejection [1, 31]. ESCs do not express MHCs and co-stimulatory molecules [32, 33] and are considered to be immune privileged [34]. In a direct comparison, our RT-qPCR analysis revealed that mESC-FBs expressed about 10 times lower levels of MHC I than BM-MSCs, while MHC II was barely detected (Fig. 3d). There were no significant changes in the expression levels of MHC I and MHC II between mESCs and mESC-FBs, indicating that in vitro differentiation methods used for the generation of mESC-FBs did not result in an apparent upregulation of MHCs.

### mESC-FBs have robust growth potential and are senescence resistant

**Fig. 4:**
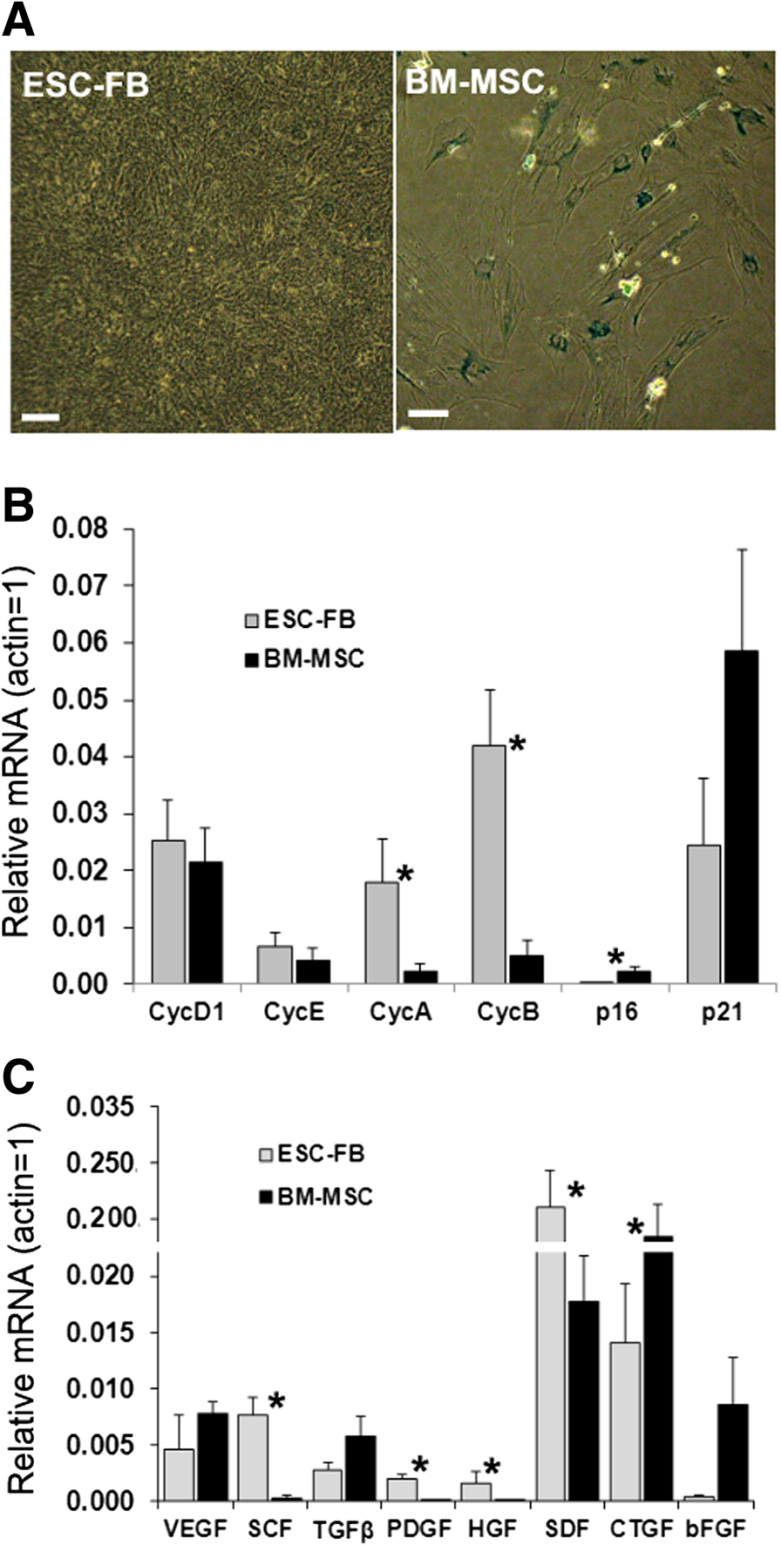
Analysis of senescence, cell cycle proteins, growth factors, and chemokines. **a** Senescence analysis of mESC-FBs and BM-MSCs cultured for 7 days. Senescent cells were identified by β-Gal activity staining (blue color) and photographed under a phase-contrast microscope. Scale bar = 100 μm. **b**, **c** RT-qPCR analysis of cell cycle proteins (**b**), growth factors, and chemokines (**c**). The mRNA levels of the tested genes were determined from untreated mESC-FBs and BM-MSCs. Results are means ± SD of three to five independent experiments. *p < 0.05, compared between two cell types

While mESC-FBs display the general properties of MSCs, they have some distinctive features. One of the most notable properties of mESC-FBs is their robust growth potential and expansion capacity. When subcultured every 3–4 days, mESC-FBs can be continuously propagated for up to 50 passages, while BM-MSCs typically can propagate for only 5–10 passages [35]. In our case, BM-MSCs began to enter senescence after passage 4. As shown in Fig. 4a, mESC-FBs grew into a compact monolayer when cultured for 7 days, with very few cells showing a senescent phenotype. Under the same conditions, BM-MSCs (p4) began to enter senescence and showed typical characteristics of senescent cells: large, flattened cell bodies and positive staining for β-Gal activity [24]. At the molecular level, BM-MSCs expressed much higher levels of cell cycle inhibitors (p16 and p21), which are also commonly used as markers of senescence [24]. Conversely, mESC-FBs expressed substantially higher levels of cyclin A and cyclin B (Fig. 4b), two cyclins which are highly expressed in mESCs and are responsible for their rapid cell proliferation [24, 36]. These results suggest that mESC-FBs partly retained the higher proliferation capacity of mESCs.

MSCs produce many trophic factors that promote angiogenesis, cell survival, and wound healing [1]. RT-qPCR analysis revealed that several trophic factors commonly expressed in BM-MSCs were detected in mESC-FBs, but the expression levels of individual factors varied somewhat in BM-MSCs and mESC-FBs (Fig. 4c). Notably, connective tissue growth factor (CTGF), a matricellular protein that has important roles in cell adhesion, proliferation, and tissue repair [37], was expressed much more highly in BM-MSCs than in mESC-FBs. Conversely, stromal cell-derived factor-1 (SDF-1), a chemokine that regulates cell migration and attracts immune cells to the inflammatory site [38], was expressed about 10 times higher in mESC-FBs than in BM-MSCs (Fig. 4c)

### mESC-FBs lack response to LPS

**Fig. 5:**
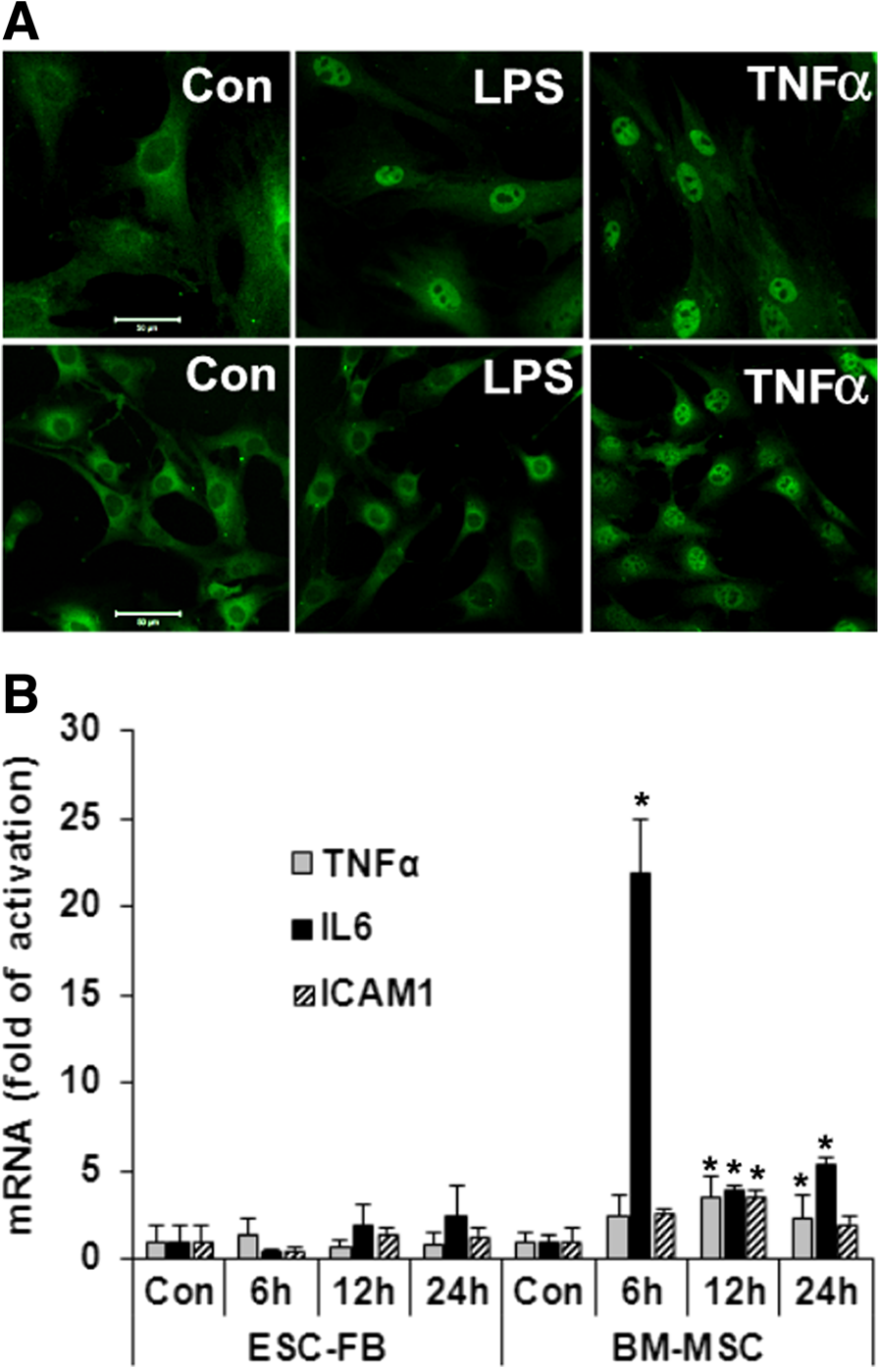
mESC-FBs do not respond to LPS. **a** BM-MSCs (upper panels) and mESC-FBs (bottom panels) were treated with LPS for 60 min or TNFα for 15 min, or left untreated (Con). The cellular location of NFκB was detected with an antibody against NFκB under a fluorescence microscope. Scale bar = 50 μm. **b** Cells were treated with LPS for the indicated time. The expression of TNFα, ICAM1, and IL6 was determined by RT-qPCR. Con represents cells without any treatment. Values are mean ± SD of samples from three independent experiments. *p < 0.05, compared with respective controls (Con)

We have previously reported that mESCs lack response to TNFα, IL1β, and LPS [9]. Here we compared mESC-FBs with BM-MSCs. Activation of NFκB transcription factor is essential in mediating the effects of the aforementioned agents. In unstimulated cells, NFκB is retained in the cytoplasm by inhibitor of NFκB protein (IκB). Upon cell activation, NFκB is translocated to the nucleus where it activates transcription of target genes. Therefore, nuclear translocation is commonly used as an indicator of NFκB activation. As shown in Fig. 5a, NFκB was detected in the cytoplasm of mESC-FBs and BM-MSCs as expected in control cells. Both LPS and TNFα induced NFκB nuclear translocation in BM-MSCs (Fig. 5a, upper panels), but only TNFα, not LPS, induced NFκB nuclear translocation in mESC-FBs (Fig. 5a, lower panels). We further analyzed the responsiveness of BM-MSCs and mESC-FBs to LPS by determining the expression of TNFα, IL6, and ICAM1, three genes that are under the transcriptional control of NFκB [39, 40]. Consistent with NFκB nuclear translocation experiments, LPS increased the expression of all three genes in BM-MSCs, but it did not significantly alter their expression level in mESC-FBs at any times tested (Fig. 5b). Therefore, the lack of response to LPS is a distinctive feature of mESC-FBs from BM-MSCs. LPS-activated signaling occurs via its interaction with TLR4 at the cell surface. Through a series of signaling events, activated TLR4 leads to NFκB activation [41]. We demonstrated that TLR4 was not detected in mESCs and differentiation could not result in its expression at the protein level [9], which explains the failure of LPS to activate NFκB and stimulate the expression of inflammatory molecules in mESC-FBs.

### mESC-FBs are less sensitive to cytotoxicity of inflammatory cytokines than BM-MSCs

**Fig. 6:**
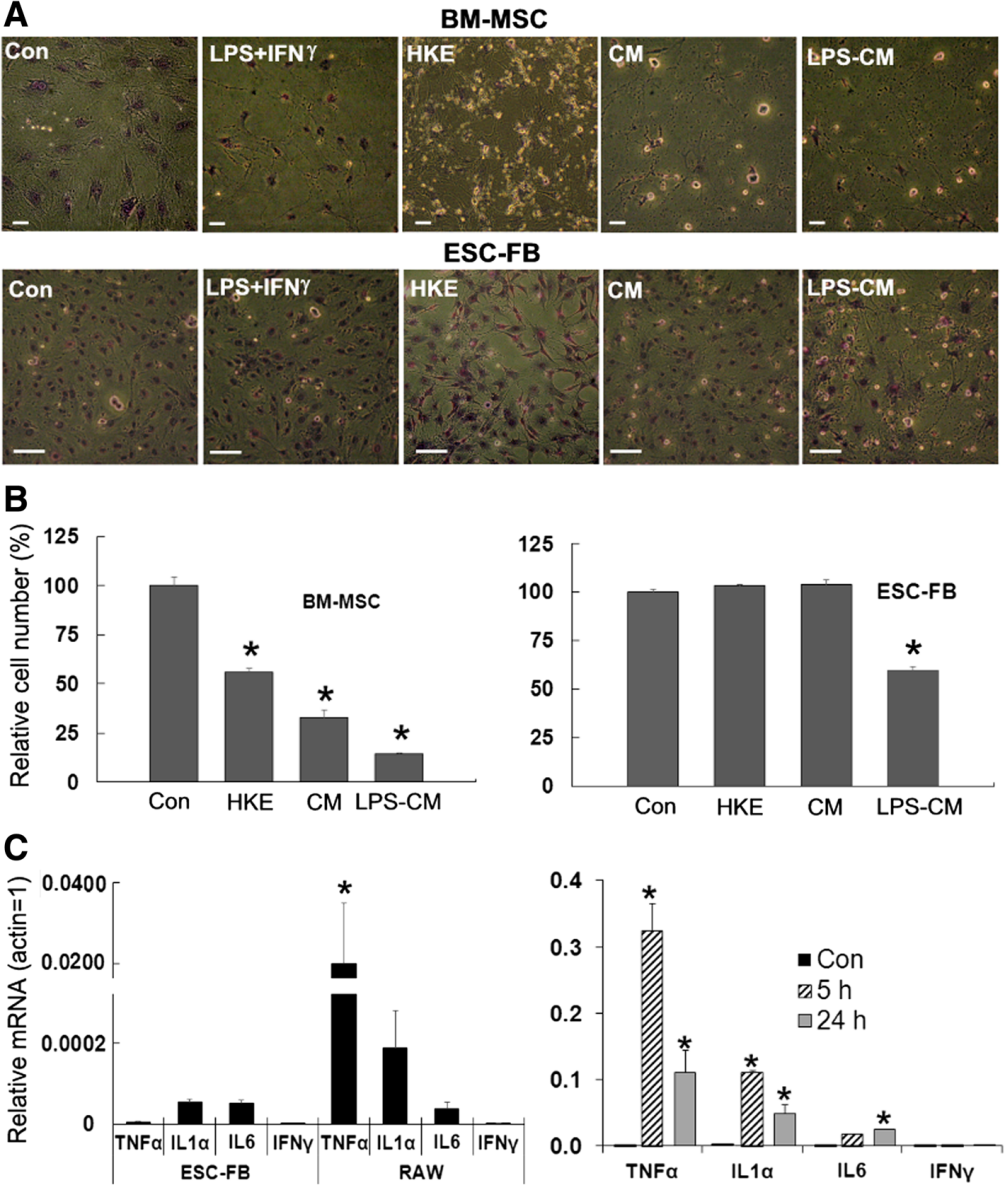
mESC-FBs are less sensitive to cytotoxicity of inflammatory conditions than BM-MSCs. **a** mESC-FBs and BM-MSCs were cultured under the indicated conditions for 4 days. The cells were fixed and stained with TB and photographed under a phase-contrast microscope, scale bar = 100 μm. **b** Quantitative analysis of relative numbers of cells under the indicated treatment conditions. Values are mean ± SD of cell numbers counted in three fields from a representative experiment under the indicated treatment conditions as described in **a**. *p < 0.05, compared with respective controls (Con). **c** RT-qPCR analysis of inflammatory cytokine expression in untreated mESC-FBs and RAW cells (left panel) and in LPS-stimulated RAW cells (right panel). Values are mean ± SD of results from three independent experiments. *p < 0.05, left panel compared between two cell types; right panel, compared with respective controls (Con)

To determine the physiological implications of lacking response to LPS in mESC-FBs, we tested the effects of several inflammatory conditions on the viability of mESC-FBs. It is known that LPS-induced inflammatory responses can cause serious cell and tissue damage if unregulated. Its cytotoxic effect can be significantly potentiated by IFNγ and the combination of LPS and IFNγ causes cell death of several types of cells [42, 43]. We treated mESC-FBs and BM-MSCs with LPS in the presence or absence of IFNγ. Neither LPS nor IFNγ alone caused detectable cell death within a period of 4 days of treatment (data not shown), but their combination caused apparent toxicity in BM-MSCs, but not in mESC-FBs (Fig. 6a). The tolerance of mESC-FBs to bacterial toxicity was further illustrated by their insensitivity to HKE, which effectively killed BM-MSCs without apparent effect on mESC-FBs (Fig. 6a). To further compare the effects of an inflammatory environment on mESC-FBs and BM-MSCs, we used an in vitro macrophage-induced inflammation model, which is based on the fact that these innate immune cells express large amounts of cytokines when activated by immunostimuli such as LPS [44, 45]. In this model, conditioned medium was collected from untreated and LPS-stimulated RAW cells (designated as CM and LPS-CM, respectively), which contained various inflammatory cytokines and immunomodulators secreted by RAW cells. Both CM and LPS-CM showed significant cytotoxicity to BM-MSCs, causing more than 50% cell death after a 4-day treatment. However, only LPS-CM, not CM, showed an apparent but much lower cytotoxic effect on mESC-FBs than on BM-MSCs (Fig. 6a). Quantitative analysis of cytotoxicity caused by HKE and RAW cell conditioned medium, as determined by the number of viable cells after treatments, is shown in Fig. 6b.

To obtain further insight into the molecules responsible for the cytotoxic effects of RAW cell conditioned medium, we examined the expression of TNFα, IL1α, and IL6 in RAW cells that were used for preparing CM and LPS-CM since these cytokines are implicated in mediating the cytotoxicity of RAW cells [46]. As expected, the expression of all three genes was stimulated by LPS in RAW cells, with TNFα being expressed at the highest level (Fig. 6c, right panel). In comparison, the basal levels of TNFα and IL1α were expressed at substantially higher levels in unstimulated RAW cells (cells used to prepare CM) than in mESC-FBs. These results could explain the cytotoxic effect of CM on BM-MSCs (as shown in Fig. 6a, b) since the cytotoxic effect of RAW cells is, at least partly, attributed to TNFα and IL1 [47, 48], which can be potentiated by LPS [45]. While we do not know what other molecules in the conditioned medium from RAW cells or HKE might contribute to the observed cytotoxic effects, our results clearly demonstrate that mESC-FBs are less sensitive than BM-MSCs to cytotoxicity caused by inflammatory conditions.

## Discussion

Ad**-**MSCs, especially BM-MSCs, have been at the forefront of developing stem cell-based therapies, but their limited availability and poor expansion capacity make them practically difficult to be used as an off-the-shelf product for therapeutic application. The generation of MSCs from both hESCs and mESCs with basic properties similar to Ad-MSCs has demonstrated the feasibility of using ESCs as an alternative source to generate MSCs [21]. The large-scale production of ESC-MSCs may overcome some limitations encountered with Ad-MSCs and offer additional valuable features for their potential therapeutic application.

To date, ESC-MSCs have been mainly characterized according to the basic criteria set for Ad-MSCs, but the unique properties of ESC-MSCs have not been well-understood [21]. The significantly higher expansion capacity of ESC-MSCs compared with BM-MSCs can be rationally explained by the high activity of telomerase in ESC-MSCs and the lack of such activity in BM-MSCs [35, 49–51]. The high levels of cyclins A and B and the low levels of cell cycle inhibitors demonstrated in this study provide additional molecular basis for the higher cell proliferation rate and senescence resistance in mESC-MSCs. In addition to the poor expansion capacity of BM-MSCs, rapid senescence as we have shown here by in vitro cell culture is another major shortfall of these cells that contributes to their transient therapeutic effect due to rapid senescence shortly after implantation [52]. Therefore, the high expansion capacity, high proliferation rate, senescence resistance, and low levels of MHC expression in ESC-MSCs could be highly desirable features with respect to therapeutic application.

The idea of using MSCs for therapeutic application was originally driven by their tri-lineage differentiation potential to repair damaged tissues. However, it is now increasingly clear that their therapeutic effects are largely attributable to their ability to secrete immunomodulators and trophic factors (collectively referred to as “bioactive factors”), which promote tissue healing and repair [1, 3]. While the expression levels of trophic factors in mESC-FBs were somewhat different from BM-MSCs, they showed a rather similar expression profile of immunomodulators in response to TNFα and IFNγ to BM-MSCs, including TNFα, IL6, iNOS, and COX-2 [30], which are among the best-characterized molecules responsible for the immunoregulatory effects of BM-MSCs [21, 30, 53]. These data suggest that mESC-FBs and BM-MSCs share basic immunoproperties. However, the immunomodulatory activity of Ad-MSCs on host immunity has become an increasingly complex issue. Studies have shown that the therapeutic effects of Ad-MSCs vary depending on their tissue origin and can be affected by the nature of the immunostimuli that lead to their activation [2, 3]. It has been proposed that LPS and polyIC could stimulate BM-MSCs to express different sets of immunomodulators, thereby promoting BM-MSCs to adopt either “pro- or anti-inflammatory phenotypes” [54]. However, inconsistent and even conflicting results have been reported in other studies [54–57]. The immunological behaviors of Ad-MSCs seem to be rather “elusive” [58]. The lack of response to LPS demonstrated in this study represents a distinctive feature of mESC-MSCs. It is conceivable that their immunobehavior and therapeutic value could significantly differ from BM-MSCs, which is an important subject that will need to be investigated with dedicated future studies.

The biological implications of underdeveloped innate immunity in ESCs can be viewed from different perspectives of stem cell biology, developmental biology, and immunology (reviewed in [59, 60]). The attenuated innate immunity in mESC-FBs is inherited from mESCs. This is not surprising considering the fact that mESC-FBs are generated by in vitro differentiation, which cannot faithfully recapitulate the differentiation process in vivo. As such, the attenuated innate immunity in mESC-FBs (or other types of ESC-differentiated cells for that matter) could be a deficiency associated with in vitro differentiation, but it could be a desirable feature for their therapeutic application. The inflammatory response has been viewed as a double-edged sword. On the one hand, it is a mechanism that defends the organism against pathogenic invasion. On the other hand, it causes various adverse effects when it is dysregulated, such as cell cycle inhibition and even cell death of infected tissues [61, 62]. From this perspective, the lack of response to LPS, which is a property inherited from mESCs [9], could allow mESC-FBs to survive better under inflammatory conditions as demonstrated by our in vitro experiments. In fact, mESCs, which also lack response to TNFα [9], are insensitive to the cytotoxic effects of inflammatory stimuli that cause cell death of BM-MSCs described in this study (unpublished results). However, it is also possible that this property could compromise their immune suppressive ability. It should be noted that previous studies have demonstrated that both soluble mediators secreted by MSCs and direct MSC interaction with immune cells, such as T cells, contribute to their immunomodulatory effects [21]. While the current study has shown that ESC-FBs resemble MSCs in producing inflammatory mediators, whether or not they exhibit immune suppressive effects on immune cells through cell-cell interaction is an important question that remains to be addressed. Therefore, how the attenuated innate immunity affects the fate, functionality, and therapeutic value of mESC-FBs is a complex issue that needs to be further investigated by in vitro studies and assessed under in vivo settings.

## Conclusion

This study demonstrated that mESC-FBs can be classified as a new class of MSCs with general properties of Ad-MSCs and additional unique features inherited from ESCs. Their robust expansion capacity, resistance to senescence, and tolerance to the cytotoxic effect of inflammatory cytokines could be desirable features for their therapeutic application. How attenuated innate immunity in ESC-MSCs will affect their immunoregulatory activity under in vivo conditions remains to be determined, but it is an attractive feature that could be modulated during in vitro differentiation based on the fact that innate immunity is developmentally regulated. By controlling differentiation conditions, it is feasible to develop a strategy that can generate ESC-MSCs with high regenerative capacity and desirable immunoproperties for therapeutic application. However, the immunological properties, especially whether ESC-MSCs show immune suppressive effect on immune cells through cell-cell interaction, will need to be further characterized by in vitro and in vivo studies.

## Abbreviations

Ad-MSC: Adult-MSC
BM-MSCs: Bone marrow-MSC
CM: Conditioned medium
ESC: Embryonic stem cell
ESC-DC: ESC-differentiated cell
FB: Fibroblast
hESC: Human ESC
HKE: Heat-killed *E.coli*
iPSC: Induced pluripotent stem cells
LPS: Lipopolysaccharide
mESC: Mouse ESC
MSC: Mesenchymal stem cell
TB: Toluidine blue

## References

[1] Ma S, Xie N, Li W (2014). Immunobiology of mesenchymal stem cells. Cell Death Differ.

[2] Bianco P, Robey PG, Simmons PJ (2008). Mesenchymal stem cells: revisiting history, concepts, and assays. Cell Stem Cell.

[3] Caplan AI (2017). Mesenchymal stem cells: time to change the name!. Stem Cells Transl Med.

[4] Nauta AJ, Fibbe WE (2007). Immunomodulatory properties of mesenchymal stromal cells. Blood.

[5] Wobus AM, Boheler KR (2005). Embryonic stem cells: prospects for developmental biology and cell therapy. Physiol Rev.

[6] Kimbrel EA, Lanza R (2015). Current status of pluripotent stem cells: moving the first therapies to the clinic. Nature Rev Drug Dis.

[7] Wang R, Wang J, Acharya D (2014). Antiviral responses in mouse embryonic stem cells: differential development of cellular mechanisms in type I interferon production and response. J Biol Chem.

[8] D'Angelo W, Acharya D, Wang R (2016). Development of antiviral innate immunity during in vitro differentiation of mouse embryonic stem cells. Stem Cells Dev.

[9] D’Angelo W, Gurung C, Acharya D (2017). The molecular basis for the lack of inflammatory responses in mouse embryonic stem cells and their differentiated cells. J Immunol.

[10] Jordana M, Sarnstrand B, Sime PJ (1994). Immune-inflammatory functions of fibroblasts. Eur Respir J.

[11] Wong T, McGrath JA, Navsaria H (2007). The role of fibroblasts in tissue engineering and regeneration. Br J Dermatol.

[12] Rajan R, Ye J, Bai S (2008). NF-kB, but not p38 MAP kinase, is required for TNF-α-induced expression of cell adhesion molecules in endothelial cells. J Cell Biochem.

[13] Foldes G, Liu A, Badiger R (2010). Innate immunity in human embryonic stem cells: comparison with adult human endothelial cells. PLoS One.

[14] Zampetaki A, Xiao Q, Zeng L (2006). TLR4 expression in mouse embryonic stem cells and in stem cell-derived vascular cells is regulated by epigenetic modifications. Biochem Biophys Res Commun.

[15] Zampetaki A, Zeng L, Xiao Q (2007). Lacking cytokine production in ES cells and ES-cell-derived vascular cells stimulated by TNFα is rescued by HDAC inhibitor trichostatin a. Am J Physiol Cell Physiol.

[16] Glaser DE, Gower RM, Lauer NE (2011). Functional characterization of embryonic stem cell-derived endothelial cells. J Vasc Res.

[17] Haniffa MA, Collin MP, Buckley CD (2009). Mesenchymal stem cells: the fibroblast's new clothes?. Haematologica.

[18] Denu RA, Nemcek S, Bloom DD (2016). Fibroblasts and mesenchymal stromal/stem cells are phenotypically indistinguishable. Acta Haematol.

[19] Chang Y, Li H, Guo Z (2014). Mesenchymal stem cell-like properties in fibroblasts. Cell Physiol Biochem.

[20] Lv FJ, Tuan RS, Cheung KMC (2014). Concise review: the surface markers and identity of human mesenchymal stem cells. Stem Cells.

[21] Gao F, Chiu SM, Motan DAL (2016). Mesenchymal stem cells and immunomodulation: current status and future prospects. Cell Death Dis.

[22] Boregowda SV, Krishnappa V, Phinney DG, Gnecchi M (2016). Isolation of mouse bone marrow mesenchymal stem cells. Mesenchymal stem cells: methods and protocols.

[23] Koziel J, Maciag-Gudowska A, Mikolajczyk T (2009). Phagocytosis of staphylococcus aureus by macrophages exerts cytoprotective effects manifested by the upregulation of antiapoptotic factors. PLoS One.

[24] Guo YL, Chakraborty S, Rajan S (2010). Effects of oxidative stress on mouse embryonic stem cell proliferation, apoptosis, senescence, and self-renewal. Stem Cells Dev.

[25] Reger RL, Tucker AH, Wolfe MR, Prockop DJ, Bunnell BA, Phinney DG (2008). Differentiation and characterization of human MSCs. Mesenchymal stem cells: methods and protocols.

[26] Pfaffl MW (2001). A new mathematical model for relative quantification in real-time RT-PCR. Nucl Acids Res.

[27] Wang R, Wang J, Paul AM (2013). Mouse embryonic stem cells are deficient in type I interferon expression in response to viral infections and double-stranded RNA. J Biol Chem.

[28] Dominici M, Le Blanc K, Mueller I (2006). Minimal criteria for defining multipotent mesenchymal stromal cells. The International Society for Cellular Therapy position statement. Cytotherapy.

[29] Penfornis P, Pochampally R. Isolation and expansion of mesenchymal stem cells/multipotential stromal cells from human bone marrow. In: Vemuri M, Chase LG, Rao MS, eds. Mesenchymal Stem Cell Assays and Applications: Humana Press, 2011:11–21. 10.1007/978-1-60761-999-4_2 21431507

[30] Ren G, Zhang L, Zhao X (2008). Mesenchymal stem cell-mediated immunosuppression occurs via concerted action of chemokines and nitric oxide. Cell Stem Cell.

[31] Caplan AI (2007). Adult mesenchymal stem cells for tissue engineering versus regenerative medicine. J Cell Physiol.

[32] Drukker M, Katz G, Urbach A (2002). Characterization of the expression of MHC proteins in human embryonic stem cells. Proc Natl Acad Sci.

[33] Magliocca JF, Held IKA, Odorico JS (2006). Undifferentiated murine embryonic stem cells cannot induce portal tolerance but may possess immune privilege secondary to reduced major histocompatibility complex antigen expression. Stem Cells Dev.

[34] Li L, Baroja ML, Majumdar A (2004). Human embryonic stem cells possess immune-privileged properties. Stem Cells.

[35] Wei H, Tan G, Manasi (2012). One-step derivation of cardiomyocytes and mesenchymal stem cells from human pluripotent stem cells. Stem Cell Res.

[36] Takumi M, Mattson M, Rao M (2008). Cellular lifespan and senescence signaling in embryonic stem cells. Aging Cell.

[37] Holbourn KP, Acharya KR, Perbal B (2008). The CCN family of proteins: structure and function relationships. Trend Biochem Sci.

[38] Takano T, Li YJ, Kukita A (2014). Mesenchymal stem cells markedly suppress inflammatory bone destruction in rats with adjuvant-induced arthritis. Lab Investig.

[39] Ledebur HC, Parks TP (1995). Transcriptional regulation of the intercellular adhesion molecule-1 gene by inflammatory cytokines in human endothelial cells: essential roles of a variant NFkB site and p65 homodimers. J Biol Chem.

[40] Libermann TA, Baltimore D (1990). Activation of interleukin-6 gene expression through the NF-kappa B transcription factor. Mol Cell Biol.

[41] Kawai T, Akira S (2007). Signaling to NF-kB by toll-like receptors. Trend Mol Med.

[42] Dijkmans R, Van Damme J, Cornette F (1990). Bacterial lipopolysaccharide potentiates gamma interferon-induced cytotoxicity for normal mouse and rat fibroblasts. Infect Immunity.

[43] So HS, Jung BH, Song HS (2001). Nitric oxide prevents the IFNgamma/LPS-induced hepatotoxicity in a protein kinase g-independent manner. Immunopharmacol Immunotoxicol.

[44] Mosser DM, Edwards JP (2008). Exploring the full spectrum of macrophage activation. Nat Rev Immunol.

[45] Cameron DJ, Churchill WH (1980). Cytotoxicity of human macrophages for tumor cells: enhancement by bacterial lipopolysaccharides (LPS). J Immunol.

[46] Ramana KV, Fadl AA, Tammali R (2006). Aldose reductase mediates the lipopolysaccharide-induced release of inflammatory mediators in RAW264.7 murine macrophages. J Biol Chem.

[47] Lee JY, Sullivan KE (2001). Gamma interferon and lipopolysaccharide interact at the level of transcription to induce tumor necrosis factor alpha expression. Infect Immun.

[48] Beutler B, Rietschel ET (2003). Innate immune sensing and its roots: the story of endotoxin. Nat Rev Immunol.

[49] Ninagawa N, Murakami R, Isobe E (2011). Mesenchymal stem cells originating from ES cells show high telomerase activity and therapeutic benefits. Differentiation.

[50] Zimmermann S, Voss M, Kaiser S (2003). Lack of telomerase activity in human mesenchymal stem cells. Leukemia.

[51] Lian Q, Lye E, Suan Yeo K (2007). Derivation of clinically compliant MSCs from CD105+, CD24- differentiated human ESCs. Stem Cells.

[52] Turinetto V, Vitale E, Giachino C (2016). Senescence in human mesenchymal stem cells: functional changes and implications in stem cell-based therapy. Int J Mol Sci.

[53] English K, Barry FP, Field-Corbett CP (2007). IFNgamm and TNFalpha differentially regulate immunomodulation by murine mesenchymal stem cells. Immunol Let.

[54] Waterman RS, Tomchuck SL, Henkle SL (2010). A new mesenchymal stem cell (MSC) paradigm: polarization into a pro-inflammatory msc1 or an immunosuppressive MSC2 phenotype. PLoS One.

[55] DelaRosa O, Dalemans W, Lombardo E (2012). Toll-like receptors as modulators of mesenchymal stem cells. Front Immunol.

[56] Zhao X, Liu D, Gong W (2014). The toll-like receptor 3 ligand, poly(I:C), improves immunosuppressive function and therapeutic effect of mesenchymal stem cells on sepsis via inhibiting MiR-143. Stem Cells.

[57] Zeuner M, Bieback K, Widera D (2015). Controversial role of toll-like receptor 4 in adult stem cells. Stem Cell Rev.

[58] Tyndall A (2014). Mesenchymal stem cell treatments in rheumatology - a glass half full?. Nat Rev Rheumatol.

[59] Guo YL, Carmichael GG, Wang R (2015). Concise reviews: attenuated innate immunity in embryonic stem cells and its implications in developmental biology and regenerative medicine. Stem Cells.

[60] Guo YL (2017). Utilization of different antiviral mechanisms by mammalian embryonic stem cells and differentiated cells. Immunol Cell Biol.

[61] Garcia MA, Meurs EF, Esteban M (2007). The dsRNA protein kinase PKR: virus and cell control. Biochimie.

[62] Samuel CE (2001). Antiviral actions of interferons. Clin Microbiol Rev.

